# Evaluation of Relationship of Neutrophil/Lymphocyte, Platelet/Lymphocyte, Monocyte/Lymphocyte Monocyte/HDL Ratios and Systemic Immune Inflammatory Index Value with Antipsychotic Treatments in Schizophrenic Patients

**DOI:** 10.1192/j.eurpsy.2024.382

**Published:** 2024-08-27

**Authors:** H. Söylemez, S. N. İspir, H. R. Demirel, E. Yıldız, M. Aydın

**Affiliations:** Department of Psychiatry, Selcuk University, Faculty of Medicine, Konya, Türkiye

## Abstract

**Introduction:**

There are studies showing that the systemic inflammation response in patients diagnosed with schizophrenia is different from healty controls. Neutrophil-lymphocyte ratio (NLR), platelet-lymphocyte ratio (TLR), monocyte-lymphocyte ratio (MLR), monocyte-HDL ratio (MHO) and systemic immune inflammation index (SII) have recently been used as inflammation indicators.

**Objectives:**

NLR, TLR, MLR, MHO and SII have been evaluated in many studies in schizophrenia patients. The aim of our study is to evaluate the relationship between NLR, TLR, MLR, MHO, SII values and antipsychotic treatments of patients diagnosed with schizophrenia.

**Methods:**

203 individuals diagnosed with schizophrenia who were followed up in the psychotic disorders outpatient clinic of Selçuk University Faculty of Medicine were included in the study. Neutrophil, lymphocyte, platelet and monocyte counts and HDL values were obtained retrospectively from blood tests. NLR, TLR, MLO, MHO and SII were calculated. The study approved by the ethics committee of Selçuk University Faculty of Medicine.

**Results:**

45.3% of the patients were female (n = 92); the mean age was 45.8±14.0. The average number of hospitalizations was 3.0±2.7 years; the mean disease duration was 17.0±9.6 years. 56.7% (n=115) use long-acting antipsychotic treatment, 21% (n=43) use monthly paliperidone long-acting (PP1M) treatment, and 14.8% (n=30) use 3-month paliperidone long-acting (PP3M) treatment. No significant difference was observed in NLR, TLR, MLR, MHO and SII values between individuals using and not using long-acting antipsychotics. However, a significant difference in NLR value was observed between PP1M and PP3M treatment (p = 0.039). Oral antipsychotic use was 71% (n=137), 19% (n=38) used clozapine monotherapy, and 25% (n=51) used non-clozapine oral monotherapy. No significant difference was detected in inflammatory markers between clozapine monotherapy and other oral monotherapies.

**Conclusions:**

According to our findings, NLR levels in patients diagnosed with schizophrenia were found to be significantly higher in those using PP1M treatment compared to those using PP3M. This finding can be interpreted in favor of the fact that PP3M contributes to the reduction of inflammation due to its longer duration of action compared to PP1M. It is thought that schizophrenia progresses through inflammatory processes and antipsychotic treatments play a role in anti-inflammation. It is envisaged that future studies may be helpful in evaluating the onset, exacerbation and remission periods of the disease, including treatment doses and durations, and revealing the relationship between inflammatory markers and schizophrenia disease and the effects of antipsychotic treatments on inflammatory markers such as NLR, TLR, MLR, MHO and SII.

**Disclosure of Interest:**

None Declared

